# Removal of Chylomicron Remnants from the Bloodstream is Delayed in Aged Subjects

**DOI:** 10.14336/AD.2017.1003

**Published:** 2018-08-01

**Authors:** Carmen G Vinagre, Fatima R Freitas, Carlos H de Mesquita, Juliana C Vinagre, Ana Carolina Mariani, Roberto Kalil-Filho, Raul C Maranhão

**Affiliations:** ^1^Heart Institute (InCor) of Medical School Hospital, University of São Paulo, São Paulo, Brazil.; ^2^University of Santo Amaro, São Paulo, Brazil; ^3^Institute of Nuclear Research, University of São Paulo, São Paulo, Brazil; ^4^Faculty of Pharmaceutical Sciences, University of São Paulo, São Paulo, Brazil

**Keywords:** Aging and atherosclerosis, chylomicron remnants, cholesterol and aging, triglyceride, emulsions

## Abstract

Dietary fats absorbed in the intestine are transported in the circulation as chylomicrons and remnants that have atherogenic potential. Although postprandial lipidemia is increased in older subjects, the specific chylomicron metabolism has not been explored in older subjects nor compared to young subjects, which is the focus of this study. After a 12 h fast, artificially-made emulsions similar to lymph chylomicrons and doubly labeled with radioactive cholesteryl esters and triglycerides were intravenously injected in 23 older (66±4 years) and 20 young (24±3 years) subjects. Sequential blood samples were collected to determine fractional clearance rates (FCR, in min^-1^) by compartmental analysis. Older subjects had higher LDL-cholesterol (p<0.001) and triglycerides (p<0.0001) than young subjects; HDL-cholesterol presented no difference. The emulsion cholesteryl-ester FCR was lower in older subjects compared to the young (p=0.0001). The emulsion triglyceride FCR did not differ in the two groups. Tested *in vitro*, however, the lipolysis of the emulsion triglycerides was less intense in the older than in the young subjects. As delayed removal of remnants, indicated by the pronouncedly smaller cholesteryl ester FCR, is related to the presence of cardiovascular diseases, this can be a risk factor which could accelerate atherogenic complications occurring in aged subjects

Chylomicrons are the lipoproteins in which dietary fats and cholesterol absorbed in the small intestine are transported in the lymph and in the bloodstream. On the endothelium surface of capillaries, chylomicron triglycerides are broken-down by lipoprotein lipase (LPL); the lipolysis products, fatty acids and glycerol are stored in adipose and muscle tissues and the resulting triglyceride-depleted remnants are finally taken-up by the liver [1, 2]. Although chylomicron remnants have atherogenic potential [3], this metabolism is difficult to evaluate. Time-consuming postprandial lipemia tests consist in the ingestion of a standard fatty meal followed by hours-long blood sampling to determine lipid marker concentration [2].

The aging process is accompanied by a rise in LDL-cholesterol due to a decrease in the capacity of the older to remove LDL [4,5]. This is probably due to a loss in the functional capacity of the LDL receptors that remove the lipoprotein from the circulation [6]. It has also been reported that postprandial lipemia [4,5] and serum concentration of retinyl palmitate [7,8] added to the test fat load as remnant labels are increased in aged subjects. The chylomicron-like emulsions method is straightforward and more precise in evaluating this metabolism, allowing for plasma kinetics analysis [9,10,11]. In this approach, artificially-made emulsions with similar composition to that of lymph chylomicrons and doubly labeled with radioactive cholesteryl esters and triglycerides are intravenously injected. Sequential blood samples are then collected to determine the decay curves of both radioisotopes and fractional clearance rates by compartmental analysis [11,12,13]. The curves of cholesteryl esters, that are not independently removed from the emulsion particles, stand for remnant removal. Meanwhile, triglyceride curves mirror the lipolysis process and the removal from the plasma of the emulsion triglycerides that were not broken-down and remain in the particles until final uptake of the remnants by the liver [11,14]. This study investigated in older subjects, as compared to young subjects, the simultaneous lipolysis and remnant removal of the chylomicron-like emulsions.

## MATERIALS AND METHODS

Twenty volunteers of both genders, aged <30years and 23 aged >60years, all healthy, were selected from the Check-up Clinics and admitted to the Lipid Metabolism Laboratory of the Heart Institute (HC-FMUSP) in São Paulo, Brazil. Participants did not have diabetes, glucose intolerance (fasting glycemia <100 mg/dL) or arterial hypertension, and were normolipidemic, non-obese and sedentary. None were under any medication, had no alcohol abuse history and they are non-smokers.

The study protocol was approved by the Ethics Committee of the University of São Paulo Medical School Hospital. An informed-signed consent was obtained from each participant.

Serum lipid analysis was performed with standard commercial kits. The chylomicron-like emulsion labeled with cholesteryl [1-^14^C] oleate (CE) and [9,10-^3^H] glycerol-tri-oleate (TG) was prepared by ultrasonic irradiation, followed by two-step ultracentrifugation, as described previously [11,12,13], and then sterilized by passage through a 0.2 micrometer filter. The emulsion was prepared in four batches; each batch was injected in subjects of the both studied groups.

**Figure 1.**
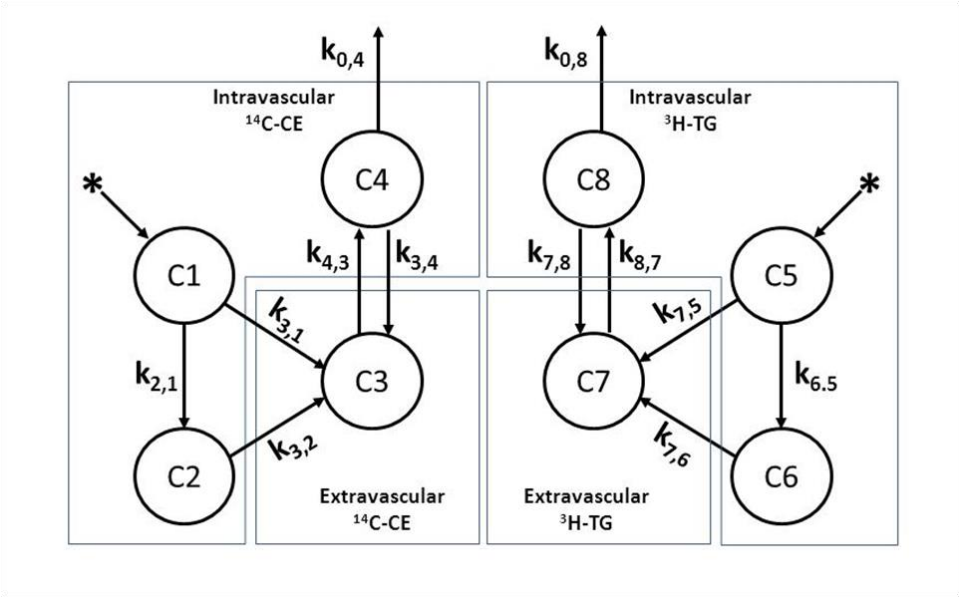
Kinetic model employed for chylomicron-like emulsion metabolism *in vivo* The observed data (compartments C1 and C2) present a biexponential curve. Compartments C1- C4 represent kinetics of ^14^C-cholesteryl oleate (^14^C-CE). Compartments C5 - C8 represent kinetics of ^3^H-triglycerides (^3^H-TG). Compartments C1 and C5 represent emulsion after plasma injection. Compartments C2 and C6 represent emulsion after redistribution. Compartments C3 and C7 correspond extra-vascular space, mainly the liver, and C4 and C8 the emulsion labeled lipids recirculation. The constants k_i,j_ (min^-1^) represent the fractional catabolic rate (FCR) or transfer from compartment *j* to compartment *i* over time. k3,1 and k7,5 - fraction of injected emulsion that is removed directly from plasma by the liver and other tissues. k2,1 and k6,5 - emulsion transfer rates to a complex plasma lipoprotein pool. k3,2 and k7,6 - removal from the plasma of the emulsion mainly by the liver. k4,3 and k 8,7 - emulsion transfer rates to VLDL (labeled lipids recirculation). k3,4 and k7,8 - recirculated lipids removal by the liver. k0,4 and k0,8 - output of labeled lipids from the body.

The volume of the emulsion to be injected into the study subjects was calculated to obtain 148 kBq (4 μCi) of ^3^H-TG and 74 (2 μCi) of ^14^C-CE. The emulsion was injected intravenously after 12 h overnight fast. Blood was collected at pre-established intervals during 60 min for radioactive counting and determination of the fractional clearance rates (FCR, in min^-1^) of the two radioactive emulsion labels by compartmental analysis model [10,11,12], according to a modification of the model proposed by Redgrave and Zech [14]. The fitting of experimental curve of the present study shows the following profile: both radioactivity decay curves (^14^C-CE and ^3^H-TG) show a rapid decay followed by a slow decay. Finally, the curve tends to a plateau out or to a smooth increase suggesting recycling of the radioactivity compounds incorporated into VLDL secreted by the liver. These features are similar to the biphasic plasma decay curve of natural chylomicrons [10]. The experimental points of plasma radioactivity measurements at the various sampling times (t, ^14^C and ^3^H) were fitted to the compartment model shown in Figure 1. The model proposed by Redgrave and Zech [14] does not consider a direct output of the compartments (C1) and (C5), but the initial absence of a plateau in the decay curve, as shown previously [10], suggests that a fraction of the injected particles is removed directly from plasma by the liver or other tissues [13]. The precision of parameters of the kinetic model presented an averaged coefficient of variation of 4.9%±10.2%.

The dose for the subjects of the radioactive material was 0.069 mSv and 0.013 for ^14^C-CE and ^3^H-TG, respectively, much lower than the permitted 1 mSv limit for radioactive intake for individuals, as determined by the International Commission on Radiological Protection [15].

*In vitro* post-heparin lipoprotein lipase (LPL) activity in the plasma was determined after 12 h fasting on the day after the kinetic studies [16,17], using as substrate a chylomicron-like emulsion labeled with glycerol tri[^3^H] oleate. The plasma samples, collected 10 minutes after an injection of heparin (10U/kg BW), and the emulsion were incubated at 37°C for pre-established intervals during 180 min. The lipids were then extracted and separated by thin-layer chromatography. The radioactivity present in the triglyceride band was measured and the area under the curve (AUC) was calculated.

Data was represented as mean±standard deviation (SD). Comparisons between two groups were assessed using the independent t-test. P values less than 0.05 were considered statistically significant.

## RESULTS

Table 1 shows the characteristics of the participant subjects of both groups. The two groups did not differ in respect to fasting glucose, BMI, and in number of male or female gender.

**Table 1:** Plasma lipids and *in vitro* lipolysis measured in subjects with age under 30 years (young group) and with more than 60 years (older group).

	Young (n=20)	Older (n=23)	P
Age (years)	24±3	66±4	<0.0001
Gender (M/F)	8/12	9/14	>0.05
BMI (kg/cm^2^)	23±3	25±4	0.8732
Glucose (mg/dL)	79±9	84±7	0.05
Cholesterol (mg/dL)			
Total	155±27	196±45	0.0010
HDL	53±11	48±12	0.1643
LDL	88±19	124±42	0.0010
Triglycerides (mg/dL)	58±24	129±56	<0.0001
*In vitro* lipolysis (AUC)	5,681±1,988	7,394±2,309	0.0133

Data expressed in mean ± SD. AUC, area under the curve.

In Table 1, it is also shown that older subjects had higher levels of LDL-cholesterol (p<0.001) and triglycerides (p<0.0001) than young subjects, whereas HDL-cholesterol presented no difference between the two groups.

Table 2 shows the compartmental parameters of the young and older groups. The emulsion cholesteryl ester FCR in older subjects was roughly 50% lower compared to the young subjects (0.008 ± 0.007 and 0.019 ± 0.010 min^-1^, respectively, p=0.0001). The emulsion triglyceride FCR did not differ in the two groups.

**Table 2:** Kinetics and compartmental analysis parameters in subjects with age under 30 years (young group) and with more than 60 years (older group).

	Young (n=20)	Older (n=23)	P
k2,1 = k6,5	0.134±9.38x10^-4^	0.145±6.45x10^-4^	<0.0001
k3,1 = k7,5	0.150±2,93x10^-4^	0.129±5.88x10^-4^	<0.0001
k3,2	0.0216±0.0097	0.0155±7.07x10^-3^	0.0222
k4,3	0.00122±0.00042	0.00470±0.00385	0.0071
k3,4	2.72x10^-6^±0.00996	0.0321±0.0079	<0.0001
k0,4	3.74x10^-7^±0.00448	0.00309±0.00369	0.0173
k7,6	0.0560±0.0007	0.0498±0.0005	<0.0001
k7,8	0.0578±0.0045	0.0422±0.0038	<0.0001
k8,7	0.00937±0.00561	0.00939±0.00471	0.9899
k0,8	4.21x10^-7^±0.00433	0.0160±0.0025	<0.0001
^14^C-CE FCR (min^-1^)	0.019±0.010	0.008±0.007	0.0001
^3^H-TG FCR (min^-1^)	0.027±0.014	0.030±0.017	0.5350

Data expressed in mean ± SD. k_i,j_ in min^-1^, compartmental parameters as indicated in Fig 1. Fractional clearance rates of the emulsion cholesteryl esters (^14^C-CE FCR) and triglycerides (^3^H-TG FCR).

The triglyceride breakdown by lipoprotein lipase, as assayed *in vitro*, was pronouncedly diminished in older group. Lipolysis was 23% less in the plasma of older subjects as compared with that of young subjects (AUC = 7,394 ± 2,309 and 5,681 ± 1,988, respectively, p=0.0001) as estimated by the AUC (Table 1).

## DISCUSSION

By means of the chylomicron-like emulsion method, this study shows that in the older subjects the removal of chylomicron remnants from the plasma is delayed when compared to young subjects, as indicated by their lower cholesteryl ester FCR. On the other hand, the chylomicron lipolysis was not diminished in older subjects, as judged by the triglyceride FCR that was the same for both the two groups. In the *in vitro* assay of post-heparin lipolysis, however, the hydrolysis of the triglycerides was smaller in the older than in the young group. The k3,1 and k7,5 in older group represent a lower removal of the emulsion by the fast component. Consequently, more emulsion particles enter the lipoprotein pool as suggested by Schwartz et al [18] and therefore increasing k2,1 and k6,5. The constants k3,2 and k7,6, that represent the slow hepatic and other tissue removal component of the decaying curve, were lower in older subjects compared to young subjects and consequently the older subjects presented slow emulsion FCR.

The issue of the plasma lipid postprandial status in the aged had been addressed in previous studies using fat load tests. In this respect, Cohn et al found correlations of the magnitude of postprandial triglyceridemia with age, in 22 subjects of both genders with ranging from 22-79 years [4]. Krasinski et al showed that in 86 men and women (19-76 years), given a fat load supplemented with retinyl ester used as chylomicron remnant label, the AUC in the plasma of the label was significantly greater in the older subjects [19], but Borel et al (1998) found no difference in the AUC in the plasma of the retinyl esters between older and young men aged 20 to 72 years [7]. In contrast, Relas et al reported that the retinyl ester AUC of eight older subjects, aged 78-79 years, was higher than that of six young subjects, aged 22-25 [8]. Puga et al found increased sequential triglyceridemia in 6 older (mean 66.7±1.1 years) when compared to 6 young (22.7±2.4 years) subjects [20]. Jackson et al, by comparing middle-age with young men (aged 46.7±1.4 and 22.3±0.9 years, respectively), found that the middle-aged had higher plasma triglyceride AUC after the ingestion of a fat load [21]. These authors separated postprandial triglyceride-rich from triglyceride-poor lipoprotein fractions and found differences in incremental triglyceridemia only in the triglyceride-rich fractions. Milan et al found similar AUC of triglyceridemia in older subjects (60-75years) and in young ones (20-25 years) after an oral fat load [22]. They noted that the peak of triglyceride values, however, was delayed in the older subjects as compared with the young subjects [22].

Tests for postprandial plasma lipids suffer from several drawbacks. Chylomicron remnants are difficult to tell apart from liver synthesized VLDLs and retinyl esters ingested to label chylomicrons partially shift from chylomicrons and remnants to other lipoprotein classes [23,24]. Apo B48 is the apo B form present in chylomicrons, whereas apo B100 is the apo B form present in VLDL and its catabolic products, is a reliable marker for remnant kinetics [24]. However, apo B48 kinetics was not performed in the previous studies focusing chylomicron metabolism and aging. At any rate, several factors that greatly differ among individual subjects and are not directly involved in plasma chylomicron metabolism, such as intestinal motility, intestinal flora interfere with fat intestinal absorption [2]. On the other hand, accumulation of VLDL triglycerides due to competition between chylomicrons and VLDL for lipoprotein lipase sites also occurs, as reported by Karpe et al [23]. All these factors introduce great variability and non-specificity referring to chylomicrons and remnants. Apo B48 determination is commercially available and is routinely measured in fasting serum. In this setting, it is difficult to ascertain whether apo B48 levels reflect chylomicron remnant catabolism, but the higher fasting levels of this protein are related with the presence of coronary artery disease [25]. The action of lipoprotein lipase is stimulated by apo C-II and inhibited by apo C-III, but it has been reported that the relation of serum apo C-II/apo C-III is not changed by aging [26].

**Figure 2.**
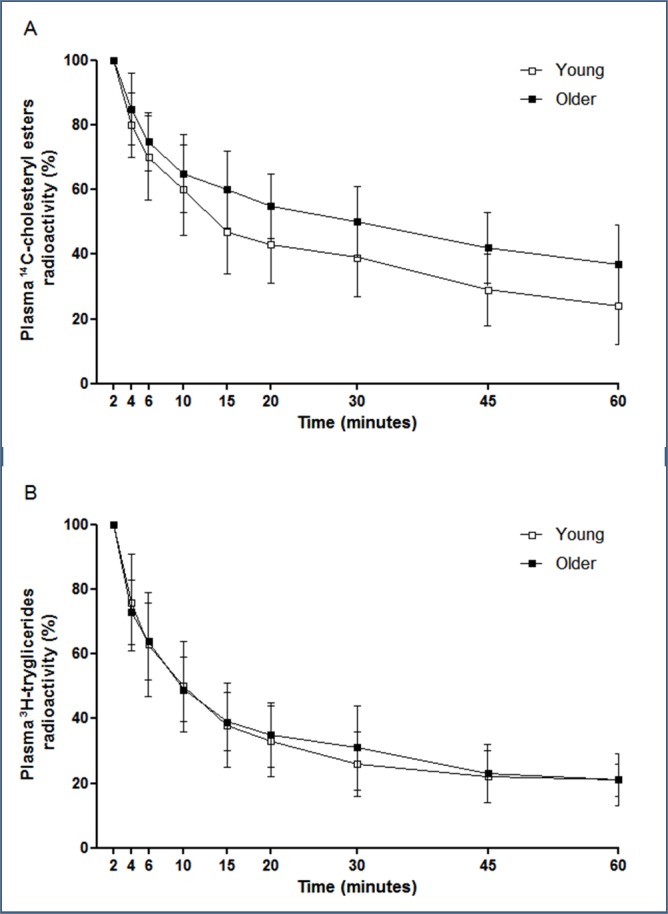
Plasma decay curve of the emulsion cholesteryl esters (A) and triglycerides (B) in healthy subjects with age under 30 years (young) and those with age above 60 years (older). The chylomicron-like emulsion labeled with the radioactive lipids was injected intravenously after a 12 hour fast. Plasma samples were taken at regular intervals over 60 minutes to determine the radioactivity remaining in the plasma in a scintillation solution.

In the chylomicron-like emulsion method, the labeled emulsions are injected into the plasma compartment, bypassing the intestinal absorption component. Injected after a 12 h fast, interfering processes such as competition with VLDL are avoided and the blood sampling period is shortened [12]. The accumulation of VLDL remnants in the postprandial state, as described by Nakajima et al [27], which interferes with the analysis of chylomicron catabolism in fat load tests, is not at play in the current experimental conditions, wherein the chylomicron-like emulsion kinetics is determined in the fast state. The emulsion method is straightforward, allowing precise calculation of the decaying parameters by compartmental analysis [10,11,12,13]. With this approach, remnants are removed from the plasma of the older subjects nearly 50% slower than in the young subjects. This is probably related with the decreased expression of LDL receptors (LDLR) in the liver that occurs in older subjects, leading to diminished LDL clearance and higher LDL-cholesterol levels [12]. LDLR do not only remove LDL but is also actively involved in chylomicron remnant uptake by the liver [28,29]. Other mechanisms are also involved in the remnant uptake by the liver. LRP1 (LDL receptor-related protein) and heparan sulfate proteoglycans (HSPGs) facilitate the rapid hepatic clearance of remnants, using apo E as ligand. Remnant particles penetrate the fenestrated endothelium in the liver and bind to lipoprotein receptors or HSPGs in the space of Disse, being subsequently internalized into the hepatocytes (for review, see Ref 6).

In this regard, we found a correlation between LDL-cholesterol and FCR of the chylomicron-like emulsion cholesteryl esters [12]. Most importantly, the fact that the residence times of native chylomicrons and chylomicron-like emulsions are similar, as shown by Park et al [30], suggests that the role of apo B48 in the uptake of remnants is not relevant. This role exerted by apo E that is present in both native and artificially-made chylomicrons.

It is of note that there was no lipolysis decrease in the older subjects, as evaluated *in vivo* by the emulsion triglyceride kinetics. However, the *in vitro* method showed that lipolysis was decreased in the older subjects compared with the young subjects, similar to the *in vitro* lipolysis results obtained by Jackson et al [21]. Since *in vitro* lipolysis is measured in post-heparin plasma, this discrepancy can be ascribed, among other causes, to age-related differences in the dissociation of lipoprotein lipase molecules from the endothelium to which the enzyme is bound by heparan sulfate bridges.

It should be pointed out that a limitation of the study was that the older group of subjects had higher LDL-cholesterol and triglycerides than the young group. This could have had influenced the emulsion kinetic results. However, it is well known that those plasma lipids systematically increase with increasing age [26]. It is also worthwhile mentioning that both groups had plasma lipids within the recommended values. Pairing the two groups for plasma lipids could then disrupt the true value of the study.

As documented by the apo B48 kinetics following a standard fatty meal [24] and by the chylomicron-like emulsion method [12], delayed removal of remnants is associated with the presence of coronary artery disease and with the evolution and clinical manifestations of this disease. Thus, the delayed remnant removal documented here by kinetic analysis may have important implications for the understanding of the atherosclerosis process that is accelerated and has become more frequently manifested in aging subjects.
